# AI-driven predictions of geophysical river flows with vegetation

**DOI:** 10.1038/s41598-024-67269-2

**Published:** 2024-07-16

**Authors:** Sanjit Kumar, Mayank Agarwal, Vishal Deshpande, James R. Cooper, Khabat Khosravi, Namal Rathnayake, Yukinobu Hoshino, Komali Kantamaneni, Upaka Rathnayake

**Affiliations:** 1 Indian Institute of Technology Patna, Patna, India; 2 University of Liverpool, Liverpool, UK; 3 Florida International University, Miami, USA; 4 University of Tokyo, Tokyo, Japan; 5 Kochi University of Technology, Kochi, Japan; 6 United Nations-SPIDER-UK Regional Support Office, University of Central Lancashire, Preston, UK; 7 University of Central Lancashire, Preston, UK; 8 Atlantic Technological University, Sligo, Ireland

**Keywords:** Flow velocity, Alluvial channel, Vegetation, Machine learning models, Empirical equations, Hydrology, Environmental impact, Civil engineering

## Abstract

In river research, forecasting flow velocity accurately in vegetated channels is a significant challenge. The forecasting performance of various independent and hybrid machine learning (ML) models are thus quantified for the first time in this work. Utilizing flow velocity measurements in both natural and laboratory flume experiments, we assess the efficacy of four distinct standalone machine learning techniques—Kstar, M5P, reduced error pruning tree (REPT) and random forest (RF) models. In addition, we also test for eight types of hybrid ML algorithms trained with an Additive Regression (AR) and Bagging (BA) (AR-Kstar, AR-M5P, AR-REPT, AR-RF, BA-Kstar, BA-M5P, BA-REPT and BA-RF). Findings from a comparison of their predictive capabilities, along with a sensitivity analysis of the influencing factors, indicated: (1) Vegetation height emerged as the most sensitive parameter for determining the flow velocity; (2) all ML models displayed outperforming empirical equations; (3) nearly all ML algorithms worked optimal when the model was built using all of the input parameters. Overall, the findings showed that hybrid ML algorithms outperform regular ML algorithms and empirical equations at forecasting flow velocity. AR-M5P (R^2^ = 0.954, R = 0.977, NSE = 0.954, MAE = 0.042, MSE = 0.003, and PBias = 1.466) turned out to be the optimal model for forecasting of flow velocity in vegetated-rivers.

## Introduction

Vegetation in an aquatic environment, such as aquatic herbs, plants, saplings, and shrubs that blossom around the water body, may be either submerged in the flow or emergent. The presence of vegetation decreases flow velocity and promotes local sedimentation by enhancing hydraulic roughness. Thus, being able to forecast accurately flow velocity is important for estimating flow resistance and the shear stress acting on the bed, and for producing estimates of flow depth and sediment transport. Nevertheless, our comprehension of the comprehensive impact of vegetation cover on river hydraulics, encompassing factors such as size, density, arrangement of vegetation stems, height of submergence, stem flexibility, geometry, and spacing, remains incomplete^1^, making flow velocity forecasting in vegetated alluvial channels a significant challenge in river science.

The velocity profiles generated by submerged and emergent vegetation differ due to a contrast in height and flexibility of the vegetation. The complexity of estimating these profiles escalates when the boundary roughness undergoes variations tied to the vegetation's growth stage, along with the temporal alignment of these changes with seasonal differences in river flows, often affecting whether the vegetation is submerged or emergent^2^. For example, Kouwen et al.^3^ performed various laboratory flume experiments and concluded that velocity profile above the vegetation layer followed the logarithmic law.

Velasco et al.^4^ performed numerous lab experiments to ascertain the flow resistance occurring due to varying densities of flexible vegetation. Their results showed the velocity profile within the canopy differed from a logarithmic profile due to the existence of vegetation stems in the flow, and that the profile shape is related to the deflected height of the plants. Wilson et al.^5^ also concluded that plant form has a significant effect on the mean flow field. A similar vertical change in flow structure was also observed by Chen et al.^6^. Their experiments showed a considerable variation in the flow field at the sheath section and at the top of a plant clump. The plants foliage thus contributes to the plant's global resistance, reaching 40% of the overall drag^7^.

Other researchers have focused on understanding how flow dynamics are impacted by the existence of vegetation. For example, Ikeda and Kanazawa^8^ conducted experiments to examine the three-dimensional, organized vortices generated above flexible vegetation. Liu et al.^9^ performed lab experiments to examine velocity profiles under rigid acrylic dowels. Their discoveries support the idea that the flow along the riverbed and atop vegetation exhibits notable instability, leading to the formation of coherent structures and significant exchange of mass and momentum.

Stoesser et al.^10^ also showed that the interspacing between the vegetation impacts turbulence by altering the 3D flow patterns. Their study found that, cylinder (or vegetation) density had a greater impact on flow and turbulence than the cylinder Reynolds number. Flow velocity in vegetated channels can be forecasted using four main types of model: theoretical, numerical/mathematical, empirical and machine-learning approaches^11^. Theoretical and numerical attempts have included using first-order and higher-order closure models^12–15^. Neary^11^ showed that reasonable forecasting of velocity profiles is achieved by adopting universal values for all model coefficients.

Choi and Kang^16^ worked on numerical simulations and found that flow quantities are optimal forecasted using Reynolds stress model as compared to others approaches. Theoretical descriptions are usually complex however, and often require poorly understood closure parameters, and at times, there are practical difficulties in collecting such data, especially in natural rivers. To overcome these difficulties, others have developed empirically based regression models to estimate depth-averaged velocity. For example Green^17^ utilized natural vegetated fields to generate percentiles of blockage factor (the fraction of a cross-section blocked by vegetation), which were then regressed against vegetation resistance. The optimal results were obtained using an exponential optimal-fit connection utilizing the 69th blockage-factor percentile.

Huthoff^18^ proposed an alternate model for flow velocity within submerged vegetation. The model was constructed based on a two-layer approach, with distinct characterizations for the flow above and through the plant layer. Other linear empirical models, developed mainly from experimental datasets, include Kouwen and Fathi-Moghadam^19^, Stephan and Gutknecht^20^, Stone and Shen^21^, Velzen et al.^22^, Huthoff^18^, and Baptist et al.^12^. These equations provide an underlying relation between flow velocity and vegetation interactions, but their applicability beyond the conditions in which they were derived and developed is limited.

In natural rivers, flow conditions depend on flow resistance and roughness type, with bedform dynamics regulating flow resistance. Manning's equation is commonly used for predicting roughness. Mir and Patel^23^ used ML models to predict Manning's roughness coefficient (n) based on six input features. Random forest, extra trees regression, and extreme gradient boosting models performed exceptionally well (R^2^ = 0.99), while Lasso Regression showed moderate efficiency. Sensitivity analysis revealed the energy grade line as a crucial predictor, providing deeper insights into riverbed characteristics and the complex relationship between roughness and other parameters.

Kouwen and Fathi-Moghadam^19^ proposed a modified model for estimating coniferous tree resistance coefficients in open-channel flow that takes into account species flexibility variations. Experiments have validated that model, which effectively incorporates vegetation-flow interactions while improving accuracy over existing methods. Key findings include a method for estimating Manning's n value, which improves flow resistance predictions in vegetated channels. Stephan and Gutknecht^20^ investigated the impact of roughness caused by submerged macrophytes on flow dynamics, emphasizing their adaptability and variable nature in various flow scenarios. Conventional flow formulas are inadequate for this complexity, necessitating the development of a hydraulic roughness parameter based on deflected plant height. Laboratory experiments with three types of aquatic vegetation revealed a relationship between hydraulic roughness and deflected plant height, resulting in a more precise quantification method.

Stone and Shen^21^ conducted extensive flume experiments to study flow hydraulics in an open channel with circular cylindrical roughness. The results showed that flow resistance varies with flow depth, stem concentration, length, and diameter and is best expressed as the maximum depth-averaged velocity between stems. They developed and validated physically based formulas for flow resistance and velocities in roughness and surface layers, which enable the calculation of channel hydraulic conditions. Velzen et al.^22^ submitted a RIZA report on floodplain vegetation flow resistance for the Directorate of Public Works and Water Management in the East Netherlands, which summarizes office studies conducted in collaboration with WL/Delft Hydraulics. The first section of the report is a manual that details flow resistance for various vegetation structures, while the second section discusses resistance formulations, vegetation structural properties, and the parameters used. The key findings include detailed descriptions and validated formulas for estimating flow resistance across various vegetation types.

Huthoff^18^ investigated methods for describing vegetation impact on flow fields, which is important for river flood studies because vegetation-covered floodplains influence flow during high discharge. It emphasizes the importance of incorporating vegetation obstruction into river-reach hydraulic models with simple, measurable input parameters that require little computational effort. The proposed method effectively meets these requirements while improving flow behavior predictions. Baptist et al.^12^ developed vegetation-induced roughness equations using a variety of methods, including two analytical methods and a numerical turbulence model. The first analytical approach simplified the vertical flow profile, whereas the second addressed the momentum balance for flow through and over vegetation. They also demonstrated the use of genetic programming to generate roughness expressions from synthetic data, which are then validated against flume experiment results. Include the effective development and validation of these roughness estimation methods.

Recently, machine learning (ML) models have been widely used to model different catchment phenomena such as floods^24^, landslides^25,26^, and incipient sediment motion^27,28^. ML methods are widely adopted these days because they able to forecast complex and non-linear environment phenomena, they require less data than other model types, are user friendly, have a non-linear structure, and without any knowledge of the underlying phenomenon, are able to formulate a non-linear and robust formula between inputs and output. Thus, these models can have a higher predictive power than both theoretical and empirical equations^28^. Data driven and ML approaches have been widely used in various hydraulic applications in rivers.

For instance, Wang et al.^29^ estimated river velocity based on GAN image enhancement and multi-feature fusion. Their results revealed ML models can produce high levels of accuracy, up to 92%. Hussain and Khan^30^ found that Random Forest models had a 17.8% and 33.6% higher performance than ANN and SVM methods for forecasting river stream flow. Others have shown that ANN models used to forecast the hydraulic geometry of irrigation canals^31^ and gravel-bed rivers^32^ outperform empirical equations. Tahershamsi et al.^33^ forecasted width of alluvial channels using multi-layer perceptron (MLP) and radial basis function (RBF) models. The performance of both models was satisfactory. Gene Expression Programming has been used to estimate bed shear stress distributions within channels, demonstrating superior performance to a well-established entropy-based model^34^.

Hybrid machine learning methods in machine learning (ML) employ the amalgamation of multiple independent ML methods to generate a more resilient predictive ML method. The aim of this method is to leverage the benefits of different base ML methods to improve the overall accuracy, robustness, and generalizability of the forecast, particularly when applied to fresh data. Hybrid machine learning methods are widely used in several fields due to their ability to tackle complex difficulties and enhance ML method accuracy.

Investigating changes in flow characteristics in open channels is crucial for understanding water ecosystems, influencing sediment deposition and water quality. Maji^35^ used Machine learning, specifically Polynomial Regression Techniques to validate laboratory experimental data of turbulent flow in a channel with emergent vegetation, showing close matches between experimental and theoretical data. Deng and Liu^36^ used a hybrid ML model, combining Bayesian Optimization with Least Squares Support Vector Machine (BO-LSSVM) to predict depth-averaged velocity in submerged vegetation flows, improving accuracy over traditional ML models and empirical formulas. Non-dimensionalization as a preprocessing method further enhances prediction performance. BO-LSSVM outperforms standalone LSSVM, SVM, and MLP models, achieving superior results and demonstrates the highest reliability in uncertainty analysis. Sensitivity analysis reveals frictional resistance parameters are more critical than bed slope parameters.

Kumar et al.^37^ evaluated multiple standalone and hybrid ML methods to predict flow velocity in vegetative alluvial channels using diverse datasets. Among the six ML methods analyzed, AR-M5P demonstrated the highest prediction accuracy. Sensitivity analysis identified vegetation height as the most critical variable in predicting flow velocity. Meddage et al.^38^ proposed models using tree-based ML models (Decision Tree, Extra Trees, XGBoost) to predict bulk-average velocity and surface layer friction factor (fS), with SHAP for interpretation. Existing regression models, despite accuracy, lack feature importance and causality insights. XGBoost outperforms in predicting bulk-average velocity (R = 0.984) and fS (R = 0.92). SHAP enhances understanding by revealing prediction rationale, dependencies, and feature importance, aligning with observed flow behaviors and increasing trust in the predictions.

Boraah and Kumar^39^ investigated the impact of vegetation on the transport of sediment and the flow of water in river channels. They discovered that aquatic plants regulate the mean flow and turbulence, reduce discharge, and increase sediment accumulation. The study employs the Group Method of Data Handling (GMDH) soft computing technique to model flow-vegetation interactions and predict flow resistance, given the limitations of traditional methods. The GMDH model efficiently optimizes predictions and emphasizes the impact of a variety of factors on the velocity profile by capturing the relationship between input and output parameters.

Barman and Kumar^40^ looked at how bank angle and floodplain vegetation emergence affect flow in compound channels. They used 45-degree and 90-degree bank angles, as well as three vegetation setups: no vegetation, fully submerged, and partially emergent. The findings indicate that vegetation has a significant impact on slopes, with steeper banks (90 degrees) experiencing higher velocity, Reynolds shear stress (RSS), and turbulent kinetic energy (TKE) resulting in greater instability. Increased vegetation emergence in floodplains exacerbates slope vulnerability, providing insights for improved hydraulic engineering and bank stability maintenance.

Arora et al.^41^ investigated flow structure changes at the interface of partially and fully vegetated sections and recommended fully vegetated sections near riverine structures for improved flow management. Partially vegetated sections show helical flow and increased turbulent kinetic energy downstream, while fully vegetated areas show more transverse flux and intermixing. These findings indicate that fully vegetated covers improve safety and effectiveness in managing flow around critical river structures.

Barman et al.^42^ used three soft computing techniques to predict flow velocity in vegetated channels. They discovered that the group method data handling (GMDH) model is better at making predictions than the optimizable Gaussian process regression (GPR) model and the ensemble tree (ET) model with Bayesian optimization. However, ET-B converges more quickly.

Barman et al.^43^ investigated flow past homogeneous and heterogeneous vegetation heights in a controlled setting, accounted for submerged and emergent vegetation cases. Barman et al.^43^ discovered that while height variations in fully submerged heterogeneous vegetation influence main channel flow, increased vegetation emergence and density significantly impact flow near the floodplain interaction zone. Near the water's surface, fully emergent cases show a dip effect with specific velocity gradients and negative streamwise Reynolds shear stress. Near the channel bed, sweep and ejection events are more common.

Despite the fact that all of this earlier research has demonstrated that ML algorithms have greater predictive capacity than conventional equations, they have yet to be used to forecast flow velocity in vegetated channels. As a result, there exists a significant gap in knowledge concerning the potential of machine learning algorithms and the identification of the most flexible and accurate algorithm.

### Research gap

1. Very limited studies have worked on prediction of flow velocity in vegetated alluvial channel
2. The application of hybrid ML methods along with the sensitivity analysis of the input parameters used is often missing from the existing studies. At times, researchers are not in a position to capture all the parameters due to various limitations. Using the sensitivity analysis researchers can get information regarding which parameters are important and which are relatively less important.
3. Using multiple datasets from the lab as well as flume ensures that a robust model is developed which incorporates the uncertainties from various data collected.

The current paper aims to address this knowledge gap by achieving the following objectives: (1) forecasting of flow velocity in vegetated alluvial channels using four types of standalone ML techniques—Kstar, M5P, reduced error pruning tree (REPT) and random forest (RF) models—in addition to eight types of hybrid ML methods; viz., Additive Regression (AR) and Bagging (BA) (AR-Kstar, AR-M5P, AR-REPT, AR-RF, BA-Kstar, BA-M5P, BA-REPT and BA-RF); (2) Compare and contrast the predictive capabilities of these proposed ML models with four frequently employed empirical equations.; and (3) Conduct a sensitivity analysis on the input combination that yields the highest forecasting accuracy.

This work is the first attempt to predict flow velocity in vegetated channels using a variety of machine learning methods. Based on simple flow and channel factors, the research offers new insights into ML techniques that might be used for precise and effective flow velocity forecasting.

## Methodology

### Proposed architecture

**Figure 1:**
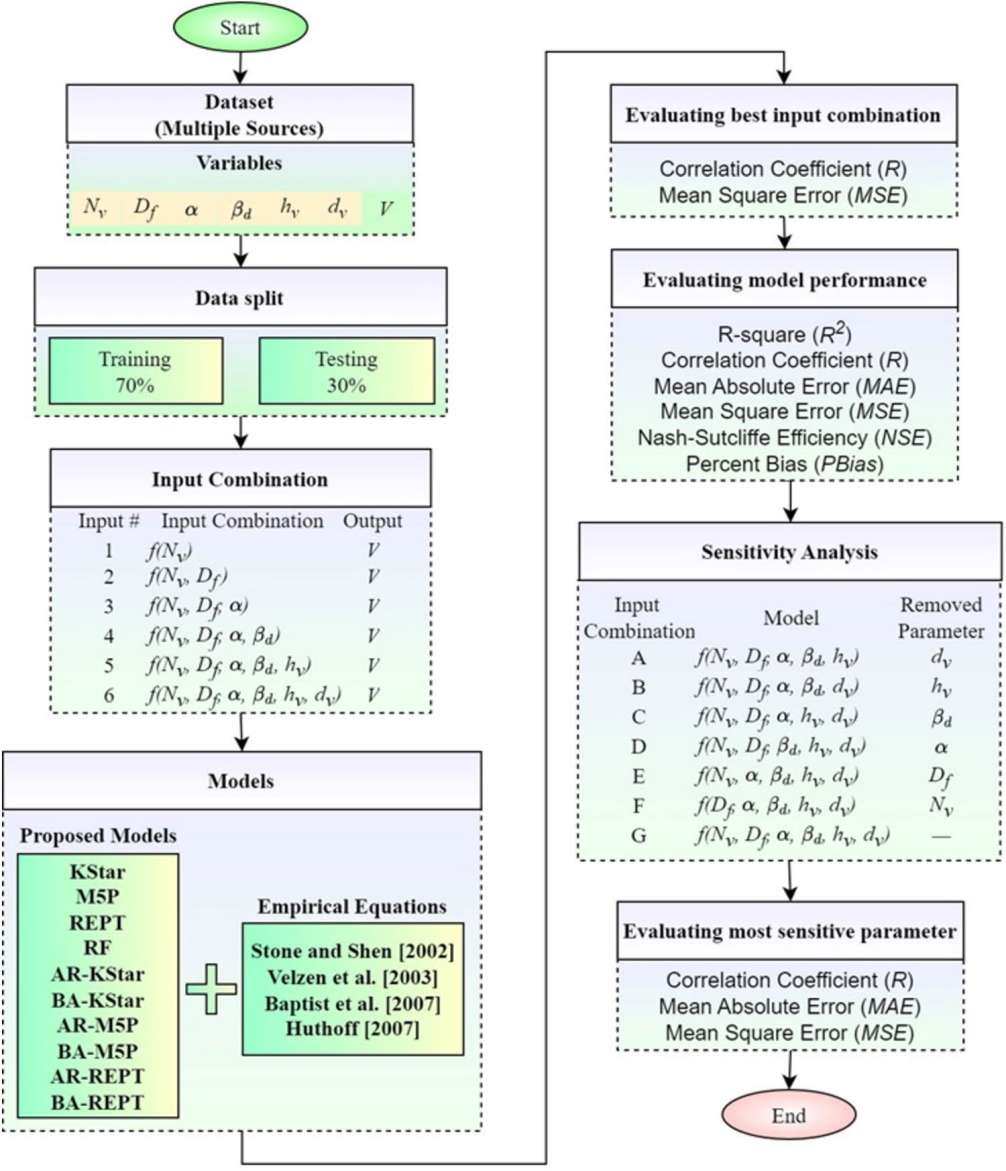
Overview of the workflow of the study.

Figure 1 presents the proposed architecture utilized in this research work for the forecasting of flow velocity. The methodology can be summarized in eight steps:

1. Data collection from different sources
2. Dimensional analysis to find the effective input parameters
3. Divide data sets for model training and testing
4. Construct different input scenarios
5. Find the effectiveness of each input parameter on the modeled results, based on sensitivity analysis
6. Develop standalone and hybrid ML approaches
7. Optimize model’s hyper-parameters
8. Compare and contrast the efficacy of the proposed models using existing approaches.

### Dimensional analysis and functional formula

Yen^1^ analyzed a number of flow resistance equations with respect to their dependent and independent parameters, revealing the following functional form can characterize flow-vegetation interactions^1^.

1 $$f(V,\alpha ,{h}_{v},{D}_{f},{N}_{v},{d}_{v},{\beta }_{d})=0$$

where *V* is the flow velocity, *α* is the channel slope, *h*_*v*_ is the height of the vegetation, *D*_*f*_ is the flow depth, *N*_*v*_ is the number of cylinders per unit vegetated area, *d*_*v*_ is the diameter of cylindrical vegetation, and *β*_*d*_ is the non-dimensional drag coefficient. Equation (1) applies to homogeneous vegetation having a fixed diameter and height of stems. The channel flow is assumed to be steady, 2D and uniform. All data comes from wide channels and thus sidewall effects are neglected^44^. In this study, *V* is viewed as a dependent variable, which mainly depends on several factors, according to Eq. (1). With this in mind, Eq. (1) can be rewritten as:

2 $$V=f(\alpha ,{h}_{v},{D}_{f},{N}_{v},{d}_{v},{\beta }_{d})$$

### Dataset

We compiled 447 data points from different sources. These datasets included Einstein and Banks^45^, Fenzl^46^, Kouwen et al.^3^, Ree and Crow^47^, Murota^48^, Tsujimoto and Kitamura^49^, Tsujimoto^50^, Tsujimoto^51^, Shimizu^52^, Dunn et al.^53^, Ikeda and Kanazawa^8^, Meijer^54^, Jarvela^55^, Rowinski and Kubrak^56^, Stone and Shen^21^, Poggi et al.^15^, Carollo et al.^57^, and Murphy et al.^58^. These studies include results for both lab-based flume experiments as well as experiments conducted on natural rivers.

After ascertaining the optimal input combination and selecting the optimal hyper-parameters, the data was split into two parts^59^ with 70% reserved for training and 30% for testing purposes. This ratio produced 314 data points for training and while 133 data points was allotted for testing phase. Table 1 presents the statistical metrics related to both the training and testing sets, as well as the entire dataset.

**Table 1:** Statistical description of the datasets.

Dataset	Parameter	Mean	Standard deviation	Minimum	Maximum
Train (count 314)	*d*_*v*_	0.006	0.004	0	0.013
	*N*_*v*_	8810.207	14,794.109	3	44,000
	*h*_*v*_	0.22	0.363	0.014	1.65
	*D*_*f*_	0.439	0.645	0.058	2.5
	α	0.005	0.008	0	0.05
	*β*_*d*_	1.245	0.562	0.67	3.14
	*V*	0.328	0.282	0.013	1.242
Test (count 133)	*d*_*v*_	0.006	0.004	0	0.013
	*N*_*v*_	8299.865	13,374.15	11	44,000
	*h*_*v*_	0.211	0.32	0.024	1.5
	*D*_*f*_	0.41	0.577	0.063	2.48
	α	0.003	0.006	0	0.044
	*β*_*d*_	1.351	0.693	0.61	3.14
	*V*	0.298	0.245	0.03	1.151
Overall (count 447)	*d*_*v*_	0.006	0.004	0	0.013
	*N*_*v*_	8658.36	14,373.294	3	44,000
	*h*_*v*_	0.218	0.35	0.014	1.65
	*D*_*f*_	0.43	0.625	0.058	2.5
	α	0.004	0.007	0	0.05
	*β*_*d*_	1.276	0.605	0.61	3.14
	*V*	0.319	0.272	0.013	1.242

### Determination of optimal input parameter combination

Six parameters (α, *h*_*v*_, *D*_*f*_, *N*_*v*_, *d*_*v*_, and *β*_*d*_) were considered as potential effective parameters. The correlation coefficient between each of these six parameters with *V* was utilized to construct different input combinations. In total, six inputs were formulated, starting with the parameter exhibiting the highest correlation with flow velocity (i.e., *N*_*v*_), followed by the inclusion of the parameter with the second highest correlation, and subsequently incorporating the parameter with the third highest correlation, continuing this sequence until all parameters were utilized (see Table 2). This approach was grounded in the assumption that parameters with the highest correlation would exert the most significant influence on forecasting power.

**Table 2:** Input combinations used in model development and testing.

Input	Input combination	Output
1	$f({N}_{v})$	*V*
2	$f({N}_{v},{D}_{f})$	*V*
3	$f({N}_{v},{D}_{f}, \alpha )$	*V*
4	$f({N}_{v},{D}_{f}, \alpha , {\beta }_{d})$	*V*
5	$f({N}_{v},{D}_{f}, \alpha , {\beta }_{d},{h}_{v})$	*V*
6	$f({N}_{v},{D}_{f}, \alpha , {\beta }_{d},{h}_{v},{d}_{v})$	*V*

### Model descriptions

#### Machine learning models

##### Kstar

The Kstar procedure^60^ is an instance-based model that was inspired by the k-Nearest Neighbor regression model. In k-Nearest Neighbor, the Euclidean metric is used to evaluate the distance between the instances, while K^*^ uses the entropy metric. The complexity of transforming instances is calculated by K^*^ distance:

3 $${K}^{*}\left(\frac{{\beta }_{k}}{{\alpha }_{k}}\right)={-log}_{2}{P}^{*}\left(\frac{{\beta }_{k}}{{\alpha }_{k}}\right)$$

where the probability of paths between instances is represented by P*. In the case of real numbers, ${P}^{*}({\beta }_{k}/{\alpha }_{k})$ depends on the difference between ${\beta }_{k}$ and ${\alpha }_{k}$.

4 $${K}^{*}\left(\frac{{\beta }_{k}}{{\alpha }_{k}}\right)={K}^{*}(j)=\frac{1}{2}lo{g}_{2}(2s-{s}^{2})-lo{g}_{2}(s)+j\left[lo{g}_{2}(1-s)-lo{g}_{2}(1-\sqrt{2s-{s}^{2}})\right]$$

where *j* = $\| {\alpha }_{k}-{\beta }_{k}\|$ and *s* is a parameter, whose value is between zero and one.

##### M5Prime (M5P)

The M5P model, proposed by Wang and Witten^61^, extends the M5 model that was initially proposed by Quinlan et al.^62^. One of the valuable features of the M5P model is that it handles large datasets consisting of a high number of features and dimensions. The model is also robust when it comes to handling missing data points in the dataset.

The M5P model initiates by partitioning the input space into multiple sub-spaces, ensuring that each sub-space encompasses data points with common features. To minimize the variability within a particular sub-space, a linear regression is used. This information is utilized to make several nodes; at these nodes a splitting process is carried out according to a given attribute. These steps help create an inverse tree-like structure with the root at the top and leaves at the bottom. When a new record comes to the system, it moves from the root, traversing the tree until it reaches the leaf node. This process helps in knowledge derivation. Model development consists of three important steps:

Step 1: To construct a tree, the input space is divided into several sub-spaces, and the specified splitting criterion is employed to minimize intra-subspace variability. In order to measure the variability, the standard deviation is used for the values that reach a node. During the M5P tree-growing procedure, the standard deviation reduction (SDR)^63^ is optimized to ensure optimal model performance. The equation for SDR is given by:

5 $$SDR = sd\left( S \right) - \mathop \sum \limits_{i} \frac{{S_{i} }}{\left\lceil S \right\rceil } \times sd\left( {S_{i} } \right)$$

where *S* represents the collection of data records that reach the node, *S*_*i*_ are the sets resulting from dividing the node based on a specified attribute, and *sd* represents the standard deviation.

- Step 2: Pruning of the tree is carried out to remove unnecessary sub-trees. This phase aims to mitigate overfitting, a phenomenon wherein a machine learning model accurately predicts training data but struggles with testing or new data.
- Step 3: The pruning process may induce sharp discontinuities between the adjacent linear models at the leaves of the pruned tree^64^. As a final stage, a smoothing process is therefore implemented to address this issue.

##### Reduced error pruning tree (REPT)

The machine learning model called the Reduced Error Pruning Tree (REPT) starts with building a decision tree and works its way up to a complete representation of the data. A pruning procedure is then used to remove superfluous branches, which avoids overfitting and enhances generalization to fresh data. After that, rules are extracted from this pruned tree, yielding a more straightforward and understandable model. The REPT model is useful in situations where precise forecasts and a thorough comprehension of the elements influencing decisions are crucial because it finds a balance between complexity and transparency.

##### Random forest (RF)

Breiman^65^ introduced a tree-based ensemble learning model RF that is used for regression as well as classification problems. In RF, multiple weak learner trees are used to compose a strong learner, so each tree is responsible for the RF errors. Multiple trees are known as forests, and if they are not fully grown, are considered deep trees. These deep trees have low bias but high variance, so they are appropriate choices for the RF model as it focuses on reducing variance. To decrease the dataset's variance, it is partitioned into numerous small subsets using a replacement method known as bootstrap sampling.

However, RF also uses another sampling method called feature sample to use a random subset of the dataset to make the tree. This method can also help in reducing the variance of the dataset. Both sample methods are introduced for RF by Dong et al.^66^ that prevent overfitting problems that can arise from multiple decision trees using the same feature to make their decision. Hence, we can say that RF model is an enhancement of bagging model with feature sample of the dataset.

##### Additive regression (AR)

Additive Regression (AR) is a ML method approach that focuses on increasing the forecasting accuracy by combining the predictions of multiple regression models. AR methods involves the creation of individual regression models for each predictor variable and then combining their outputs. The AR method aims to utilize the additive effects of each predictor on the response variable. AR models usually perform well when predictor variables interact nonlinearly, as they possess the flexibility to model complex relationships. The final model is an additive composition of these individual regressions models, providing a comprehensive representation of the overall relationship between predictors and the response variable.

##### Bootstrap aggregation (bagging/BA)

Bootstrap aggregation (bagging) is an ensemble methodology used for both regression and classification problems. In many cases, decision tree models suffer from high variance, which can be circumvented by the Bagging approach. Bagging is usually applied when the amount of data is limited, and a robust estimate of a statistical feature is required. The model uses multiple random training data samples to train multiple models for forecasting. To provide a reliable forecast, the forecasting accuracy of each of these many models is evaluated, and the averaged findings are used. By reducing the effect of individual model variances, this averaging strategy improves the forecasts' overall reliability.

For a given set of *k* independent observation *k*_*1*_*, k*_*2*_*, …, k*_*n*_ each having variance ${\sigma }^{2}$, the variance of mean *K* of the set of observation is ${\sigma }^{2}/k$. Thus by taking the average value, the resultant observed variance is reduced, and increasing the size of the training sample reduces the variance, enhancing the forecasting accuracy For sample training sets *C*, Multiple models are produced sample training sets *C.* ${f}_{1}{\prime}(x),{f}_{2}{\prime}(x),{f}_{3}{\prime}(x),..,{f}_{C}{\prime}(x),$ where $x<k$. These algorithms are averaged to obtain a low variance model:

6 $${f}_{avg}\left(x\right)=\frac{1}{C}\sum_{c=1}^{C}{f}_{b}(x)$$

However, in many instances large sample sizes are not available. To overcome this, bootstrapping is used to randomly sample multiple datasets and the averaged model is given by:

7 $${f}_{bag}\left(x\right)=\frac{1}{C}\sum_{c=1}^{C}{f}_{b}^{*}(x)$$

#### Empirical equations

The proposed approach is compared to four commonly used empirical equations (Eqs. 8–11) (Huthoff^18^; Velzen et al.^22^; Baptist et al.^12^; Stone and Shen^21^):

8 $$V=\sqrt{\frac{2g}{{\beta }_{d}{N}_{v}{d}_{v}}}\sqrt{i}\left(\sqrt{\frac{{h}_{v}}{{D}_{f}}}+\frac{{D}_{f}-{h}_{v}}{{h}_{v}}{\left(\frac{({D}_{f}-{h}_{v})\sqrt{{N}_{v}}}{1-{d}_{v}\sqrt{{N}_{v}}}\right)}^{2/3}\right)$$

9 $$V=\sqrt{\frac{2g}{{\beta }_{d}{N}_{v}{d}_{v}}}\sqrt{\alpha }+18({D}_{f}-{h}_{v}{)}^{3/2}\frac{\sqrt{\alpha }}{{h}_{v}}\mathit{log}\frac{12({D}_{f}-{h}_{v})}{1.6{{h}_{v}}^{0.7}}$$

10 $$V=\left(\sqrt{\frac{2g}{{\beta }_{d}{N}_{v}{d}_{v}}}\sqrt{\alpha }\sqrt{{h}_{v}}+\frac{\sqrt{g}}{0.4}ln\left(\frac{{D}_{f}}{{h}_{v}}\right)\sqrt{\alpha }\right)\sqrt{{D}_{f}}$$

11 $$V=\sqrt{\frac{2g}{{\beta }_{d}{N}_{v}{d}_{v}}}\sqrt{\alpha }(1-{d}_{v}\sqrt{{N}_{v}})\sqrt{\left(\frac{{D}_{f}}{{h}_{v}}-\frac{1}{4}\pi {N}_{v}{{d}_{v}}^{2}\right)\frac{{D}_{f}}{{h}_{v}}}$$

### Model performance metrics

To evaluate the effectiveness of the proposed models in forecasting the mean velocity of flow in a vegetated channel, the following six metrics were used: R^2^, R, MAE, MSE, NSE, and PBias. Their mathematical formulation is given below:

12 $$R^{2} = \left( {\frac{{\sum\limits_{i = 1}^{N} {\left( {\hat{V}_{i} - \overline{\hat{V}}} \right)} \left( {V_{i} - \overline{V}} \right)}}{{\sqrt {\sum\limits_{i = 1}^{N} {\left( {\hat{V}_{i} - \overline{\hat{V}}} \right)^{2} } } \sqrt {\sum\limits_{i = 1}^{N} {\left( {V_{i} - \overline{V}} \right)^{2} } } }}} \right)^{2} ,\quad 0 \le R^{2} \le 1$$

13 $$R = \frac{{\sum\limits_{i = 1}^{N} {(\hat{V} - \overline{\hat{V}})} (V - \overline{V})}}{{\sqrt {\sum\limits_{i = 1}^{N} {(\hat{V} - \overline{\hat{V}})^{2} } } \sqrt {\sum\limits_{i = 1}^{N} {(V - \overline{V})^{2} } } }},\quad - 1 \le R \le + 1$$

14 $$NSE = 1 - \sqrt {\frac{{\sum\limits_{i = 1}^{N} {\left( {\hat{V}_{i} - V_{i} } \right)^{2} } }}{{\sum\limits_{i = 1}^{N} {\left( {\hat{V}_{i} - \overline{V}} \right)^{2} } }}} ,\quad - \infty \le NSE \le 1$$

15 $$MAE = \frac{1}{N}\sum\limits_{i = 1}^{N} {\left( {\left| {\hat{V}_{i} - V_{i} } \right|} \right)} ,\quad 0 \le MAE \le + \infty$$

16 $$MSE = \frac{1}{N}\sum\limits_{i = 1}^{N} {\left( {\hat{V}_{i} - V_{i} } \right)^{2} } ,\quad 0 \le MSE \le + \infty$$

17 $$PBias = 100*\frac{{\sum\limits_{i = 1}^{N} {\left( {\hat{V}_{i} - V_{i} } \right)} }}{{\sum\limits_{i = 1}^{N} {\hat{V}_{i} } }}$$

where $\hat{V}$ and *V* refer to the forecasted and actual values, $\overline{\hat{V}}$ and $\overline{V}$ denote the mean forecasted and mean actual value, respectively, and *N* is the total number of data points used in the study. Ideal values of *R*^*2*^, *R*, *NSE*, *MAE*, *MSE* and *Pbias* are 1, 1 or − 1, 1, 0, 0 and 0 respectively. Model performance can be classified using the *NSE* values (between − ∞ and 1; Moriasi et al.^67^): (i) unsatisfactory: *NSE* ≤ 0.4; (ii) acceptable: 0.40 < *NSE* ≤ 0.50; (iii) satisfactory: 0.50 < *NSE* ≤ 0.65; (iv) good: 0.65 < *NSE* ≤ 0.75; (v) very good: 0.75 < *NSE* ≤ 1.00.

For visual examination Taylor diagrams, box plots as well as line and scatter plots were utilized in this study. The Taylor diagram offers the advantage of incorporating two primary correlation statistics: standard deviation (SD) and correlation ®, providing a comprehensive visualization of model performance^68^. The reference point for a Taylor diagram refers to the measured data point. The stronger the forecasting capability of a given model, the nearer the forecasted value to the reference value in terms of R and SD. A box plot’s can demonstrate how effectively a model predicts values at the extremes, median, and quartile ranges; the closer the quartile line of the forecasted value to the actual quartile, and more generally, the greater the similarity in box-plot shape, the better the model performance.

## Results

### Ascertaining the optimal input parameter combination

**Figure 2:**
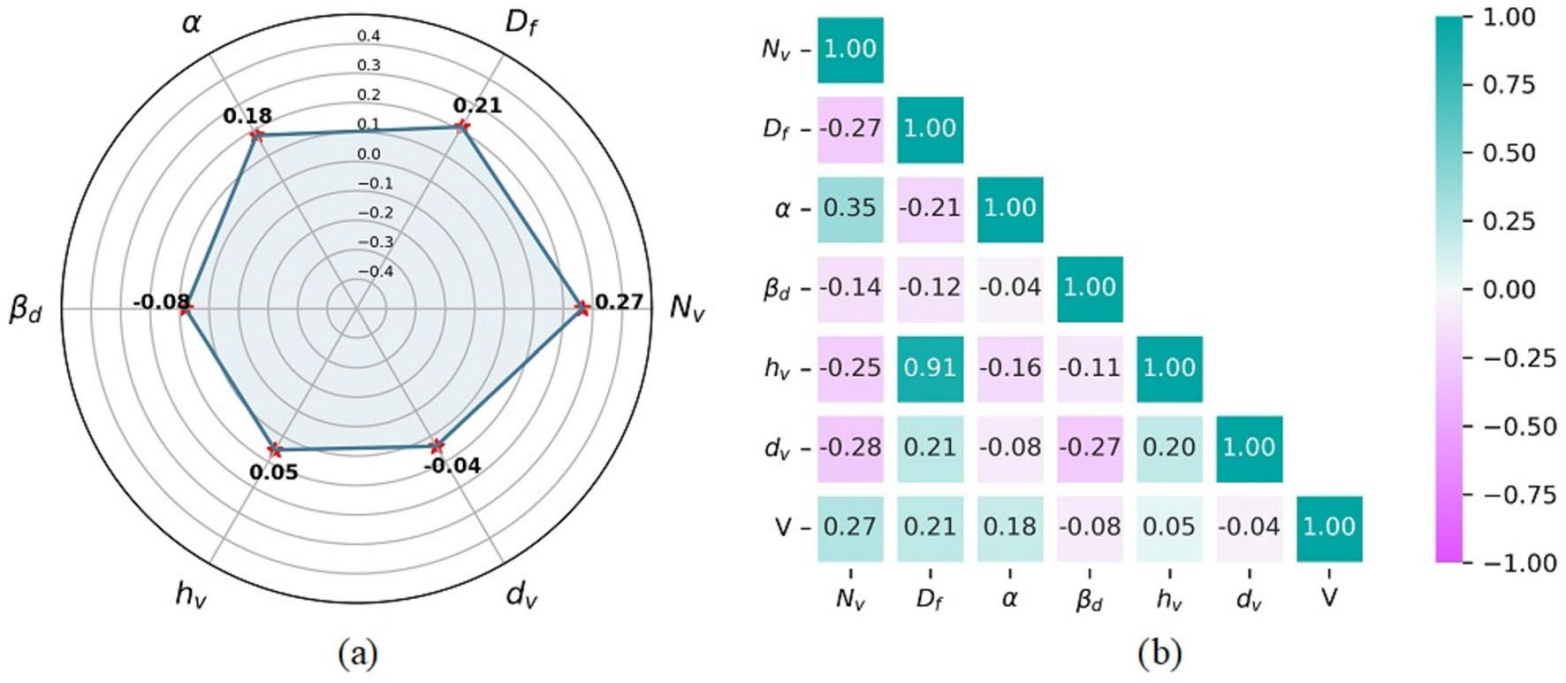
(**a**) Spider and (**b**) heat map plots illustrating the correlation coefficient between input parameters and flow velocity.

Spider and heat map plots of the correlation coefficient in Fig. 2 shows that the number of cylinders per unit vegetated area had the highest impact on flow velocity (*R* = 0.27), followed by flow depth (*R* = 0.21), channel (*R* = 0.18), non-dimensional drag coefficient (*R* = − 0.08), height of the vegetation (*R* = 0.05), and diameter of cylindrical vegetation (*R* = − 0.04).

Table 3 shows the different input combination effectiveness, based on the *R* and *MSE* values. Input 6 (all input parameters involved) was the optimal combination for seven models out of 12 models (AR-M5P, AR-REPT, AR-RF, BA-Kstar, BA-M5P, BA-RF, and RF). Input 5 (all involved except *d*_*v*_) was optimal for four models (AR-Kstar, BA-REPT, Kstar, and M5P), and the REPT model performed most strongly with Input 4 (all involved except of *d*_*v*_ and *h*_*v*_).

**Table 3:** Ascertaining the optimal input combination using model evaluation criteria.

Models	Evaluation criteria	Input 1	Input 2	Input 3	Input 4	Input 5	Input 6
AR-Kstar	*R*	0.764	0.834	0.905	0.918	**0.961**	0.958
	*MSE*	0.025	0.019	0.011	0.01	**0.005**	0.005
AR-M5P	*R*	0.758	0.845	0.882	0.892	0.965	**0.977**
	*MSE*	0.026	0.018	0.014	0.013	0.004	**0.003**
AR-REPT	*R*	0.798	0.811	0.857	0.882	0.905	**0.909**
	*MSE*	0.022	0.022	0.016	0.014	0.011	**0.011**
AR-RF	*R*	0.793	0.871	0.923	0.94	0.966	**0.97**
	*MSE*	0.023	0.015	0.009	0.007	0.004	**0.004**
BA-Kstar	*R*	0.749	0.834	0.886	0.896	0.944	**0.946**
	*MSE*	0.027	0.019	0.013	0.012	0.007	**0.006**
BA-M5P	*R*	0.754	0.845	0.895	0.899	0.957	**0.959**
	*MSE*	0.026	0.018	0.013	0.012	0.007	**0.007**
BA-REPT	*R*	0.779	0.835	0.88	0.9	**0.92**	0.915
	*MSE*	0.024	0.019	0.014	0.012	**0.01**	0.01
BA-RF	*R*	0.794	0.853	0.902	0.914	0.944	**0.946**
	*MSE*	0.023	0.017	0.011	0.01	0.007	**0.007**
Kstar	*R*	0.75	0.84	0.897	0.913	**0.953**	0.95
	*MSE*	0.027	0.018	0.012	0.01	**0.006**	0.006
M5P	*R*	0.758	0.845	0.882	0.892	**0.947**	0.935
	*MSE*	0.026	0.018	0.014	0.013	**0.007**	0.01
REPT	*R*	0.798	0.811	0.862	**0.882**	0.879	0.875
	*MSE*	0.022	0.022	0.016	**0.014**	0.014	0.014
RF	*R*	0.796	0.866	0.908	0.927	0.946	**0.956**
	*MSE*	0.023	0.016	0.011	0.009	0.006	**0.005**

Significant values are given in bold.

### Model performance

**Figure 3:**
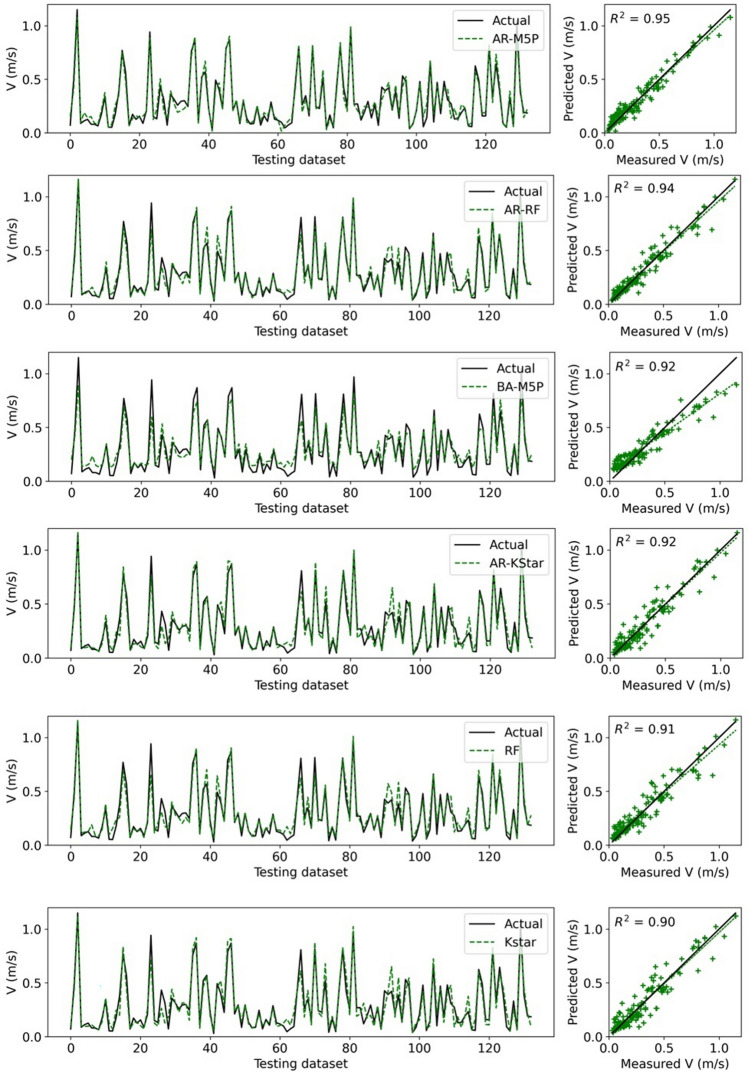

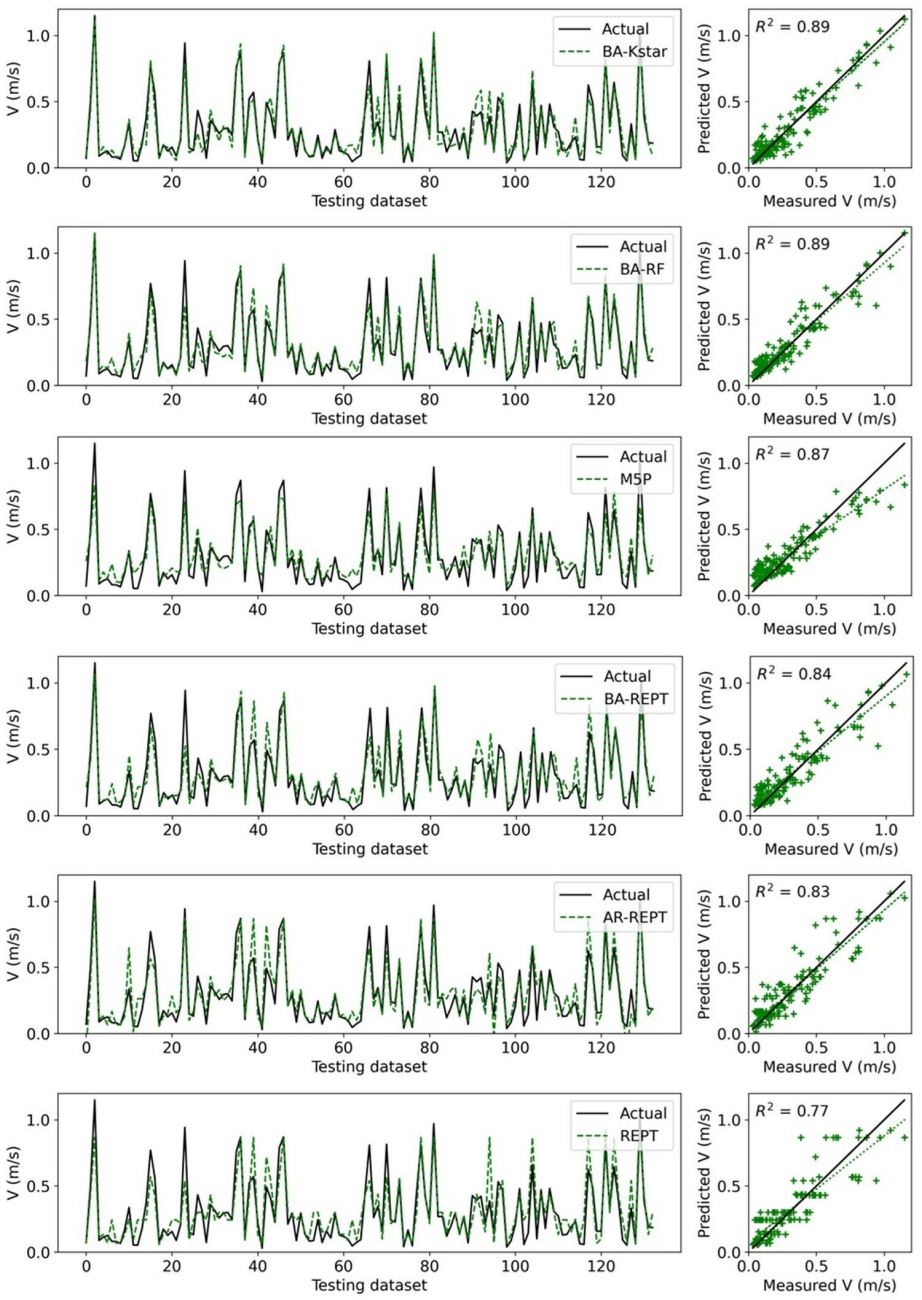
Forecasted versus actual flow velocity for the testing dataset.

Using the testing dataset, it can be observed that all ML models exhibit high performance (Fig. 3), and hybrid models are more capable than standalone models at capturing extreme values (minimum and maximum *V* values).

To benchmark this performance, Table 4 shows a comparison in performance metrics of the twelve ML models with four empirical equations. In all cases, the model performance is far superior for the ML models. All the models except the empirical equations demonstrate very good forecasting capabilities in terms of *R*^2^ (*R*^2^ > 0.7). Based on the NSE model performance classification proposed by Moriasi et al.^67^, all ML models performed very well, while empirical equations had unsatisfactory performance.

**Table 4:** Performance of applied models (bold indicates the optimal performance).

Model	*R*^*2*^	*R*	*NSE*	*MAE*	*MSE*	*PBias (%)*
AR-M5P	**0.954**	**0.977**	**0.954**	**0.042**	**0.003**	1.466
AR-RF	0.941	0.97	0.941	0.043	0.004	0.587
BA-M5P	0.92	0.959	0.878	0.067	0.007	2.619
AR-Kstar	0.917	0.958	0.915	0.052	0.005	0.267
RF	0.913	0.956	0.912	0.052	0.005	2.551
Kstar	0.902	0.95	0.899	0.058	0.006	**0.194**
BA-Kstar	0.895	0.946	0.893	0.061	0.006	0.385
BA-RF	0.894	0.946	0.891	0.059	0.007	4.072
M5P	0.875	0.935	0.839	0.078	0.01	3.374
BA-REPT	0.838	0.915	0.833	0.074	0.01	5.452
AR-REPT	0.826	0.909	0.82	0.077	0.011	2.219
REPT	0.766	0.875	0.76	0.086	0.014	5.054
Huthoff^18^	0.416	0.645	− 189.828	2.053	11.485	687.501
Velzen et al.^22^	0.36	0.6	− 17.066	0.5	1.087	146.431
Baptist et al.^12^	0.319	0.565	− 0.009	0.156	0.061	− 47.076
Stone and Shen^21^	0.285	0.534	− 0.488	0.186	0.09	− 33.689

Significant values are given in bold.

The PBias metric shows the level of bias in model performance. The optimal value of *PBias* is 0. Usually, the value of *PBias* ≤  ± 10 corresponds to very good model performance^69^. A positive PBias indicates an underestimation, while a negative PBias signifies overestimation. Although all ML models have a very good performance, Table 4 shows the *PBias* values for the standalone and hybrid version of the Kstar model are close to zero. All models, except the empirical equations of Baptist et al.^12^ and Stone and Shen^21^, demonstrate that the developed models underestimated flow velocity.

The comparison in Table 4 also reveals which of the models had the highest performance. For all metrics but *PBias*, AR-M5P model had the highest forecasting power. In the case of *PBias*, the Kstar model was judged as the optimal performing model. For all metrics the hybridized ML models outperformed their standalone counterpart.

**Figure 4:**
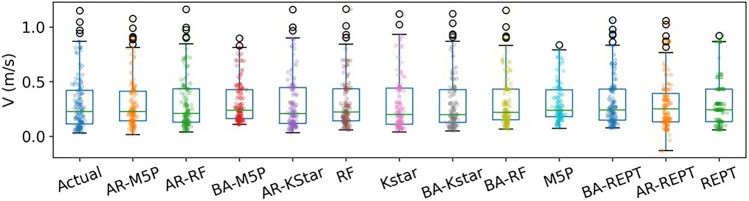
Box plot of the actual and forecasted flow velocity values by ML models.

Box plots are presented to compare the performance of both standalone and hybridized machine learning models (Fig. 4). The results show the quartiles of the AR-M5P and observed data almost coincide. In contrast, the quartile for AR-REPT shows higher deviation, indicating low performance. In terms of the maximum *V* value, the RF model and its hybridized versions (AR-RF, BA-RF) showed higher performance, while AR-M5P more accurately captured the lowest *V* value than the other models.

**Figure 5:**
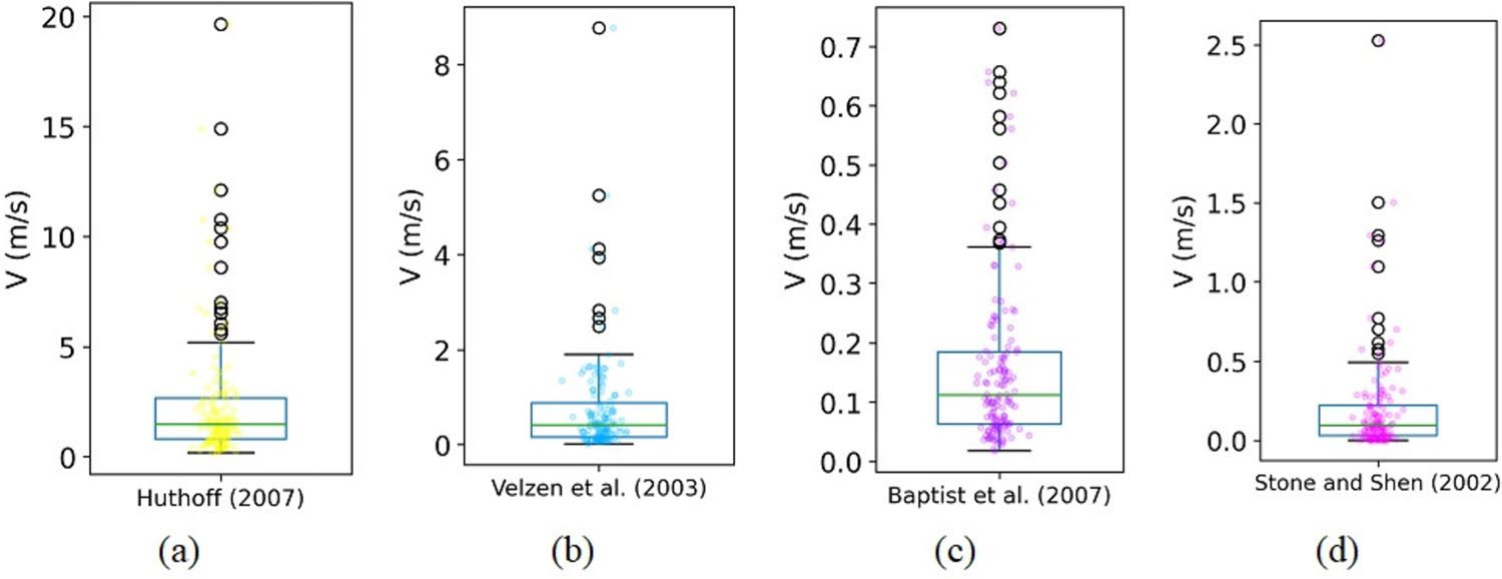
Box plots of the actual and forecasted flow velocity by four empirical equations.

Figure 5a–d shows the box plots of forecasted flow velocity for the empirical equations, plotted separately to those in Fig. 4 because they overestimate flow velocity by a very large margin. The equation developed by Baptist et al.^12^ performed better than the other empirical equations, but none of these equations were able to forecast *V* accurately.

**Figure 6:**
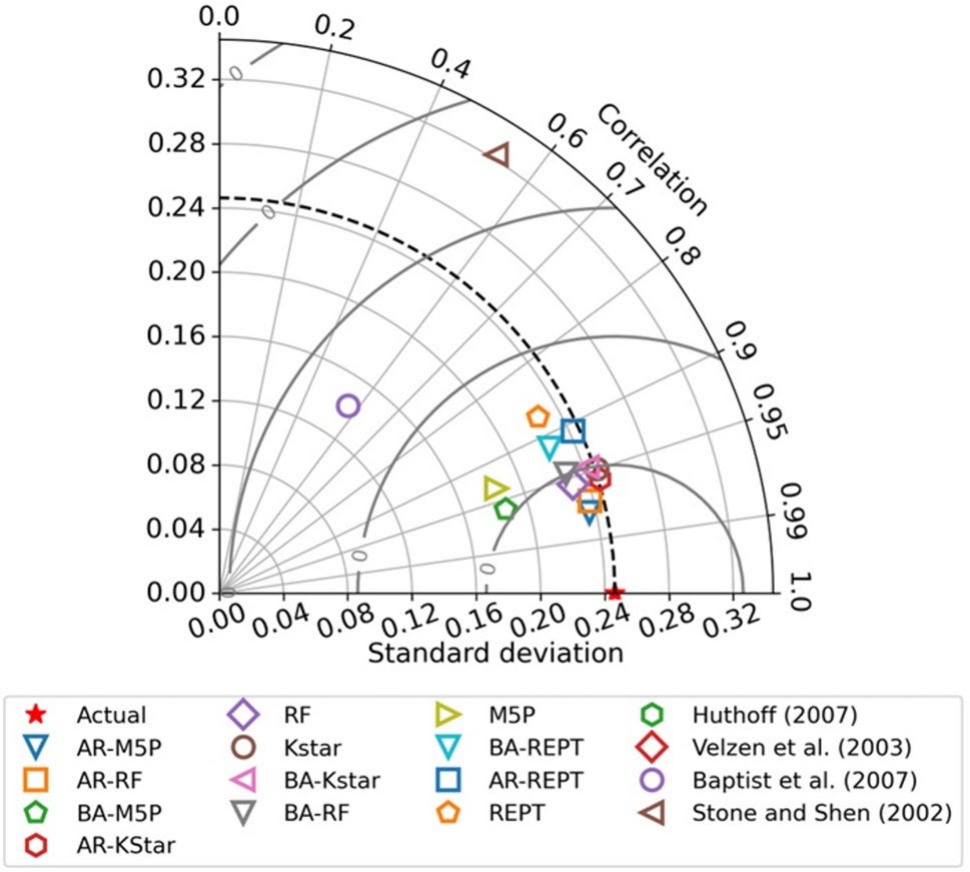
Taylor diagram illustrating model performance.

In the Taylor plot (Fig. 6), the AR-M5P model was in close proximity to the observed reference point, indicating that the forecasted standard deviation of flow velocity closely matched the observed data standard deviation, and the correlation was highest among the models evaluated. On the Taylor plot, the RA-KStar, BA-KStar, AR-RF, and BA-REPT data points nearly coincide, indicating comparable model performance. Stone and Shen’s^21^ empirical equation had the lowest performance.

### Sensitivity analysis

**Figure 7:**
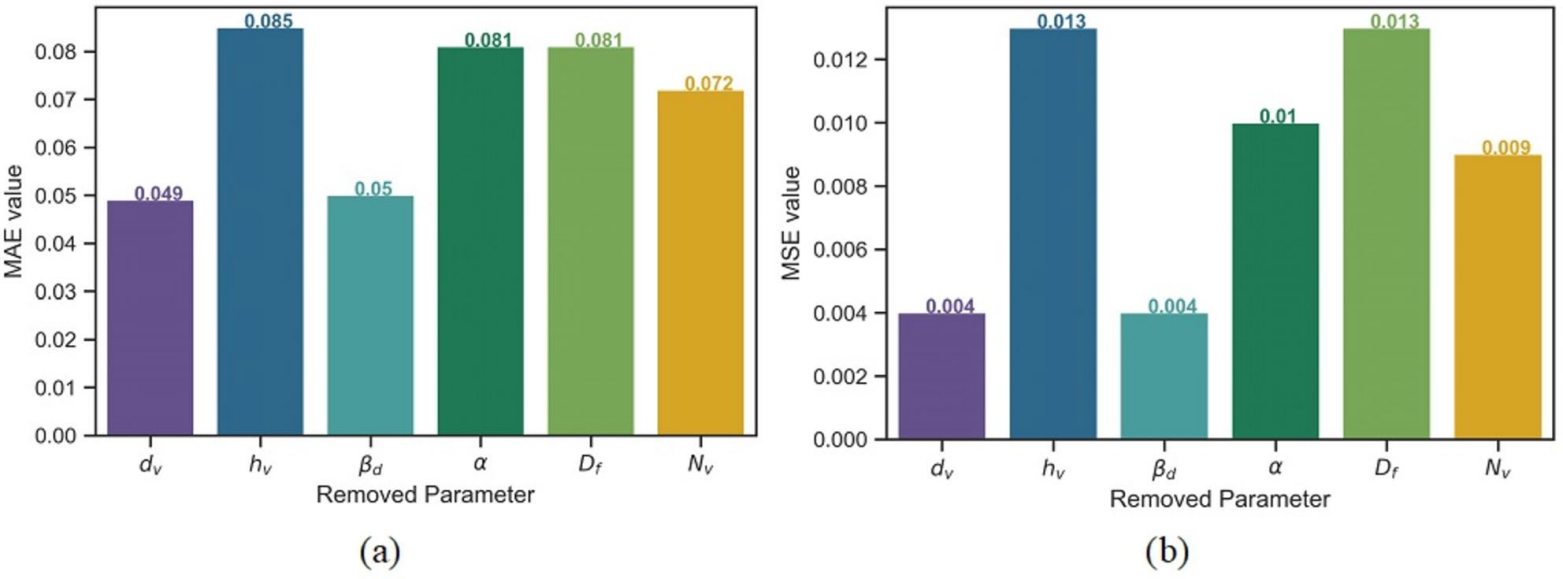
Bar chart showing difference in model performance for different input parameter combinations based on (**a**) *MAE*, and (**b**) *MSE.*

A sensitivity analysis is undertaken to understand the impact of each input parameter on flow velocity by removing one by one a parameter from the model construction and evaluating the effect on model performance. The input combinations for sensitivity analysis are shown in Table 5. For example, Input combination A removed the parameter *d*_*v*_ and used the remaining five parameters (*N*_*v*_, *D*_*f*_, *α*, *β*_*d*_, and *h*_*v*_), Input combination B removed parameter *h*_*v*_ and so on. The removal of the *h*_*v*_ parameter from the input variable combination produced the largest increase in MAE and MSE values, and thus improvement in model performance, compared to the other parameters (Fig. 7). Therefore, the *h*_*v*_ parameter was the most sensitive and effective input parameter for the forecasting of flow velocity, followed by *D*_*f*_*, α, N*_*v*_*, β*_*d*_, and *d*_*v*_.

**Table 5:** Input combinations used in sensitivity analysis.

Input combination	Model	Removed parameter
A	*f(N*_*v*_*, D*_*f*_*, α, β*_*d*_*, h*_*v*_*)*	*d*_*v*_
B	*f(N*_*v*_*, D*_*f*_*, α, β*_*d*_*, d*_*v*_*)*	*h*_*v*_
C	*f(N*_*v*_*, D*_*f*_*, α, h*_*v*_*, d*_*v*_*)*	*β*_*d*_
D	*f(N*_*v*_*, D*_*f*_*, β*_*d*_*, h*_*v*_*, d*_*v*_*)*	*α*
E	*f(N*_*v*_*, α, β*_*d*_*, h*_*v*_*, d*_*v*_*)*	*D*_*f*_
F	*f(D*_*f*_*, α, β*_*d*_*, h*_*v*_*, d*_*v*_*)*	*N*_*v*_
G	*f(N*_*v*_*, D*_*f*_*, α, β*_*d*_*, h*_*v*_*, d*_*v*_*)*	–

## Discussion

### Compare and contrast of the efficacy of empirical, standalone, and proposed hybrid machine learning models

The paper used numerous datasets collected from various sources, in which flow velocity had been measured in differing ways in vegetated channels in varied natural and laboratory conditions, to investigate the efficiency of each model. The empirical equations performed poorly, confirming these relations should be used with due caution outside the conditions for which they were developed. In contrast, all ML models performed well because they can learn and adapt to the changing data.

Among the standalone models, the RF model had a superior performance as compared to the other models. This result occurred for a number of reasons: (1) RF is better at handling datasets that contain null or missing values; (2) each constructed base tree is independent of the others, exhibiting the feature of parallelization; (3) the algorithm is extremely stable, since the average response of a large number of trees are used; and (4) the model preserves variety since all qualities are not evaluated when creating each base tree. This feature has the added advantage of minimizing the feature space and resulting in RF being unaffected by the curse of dimensionality (When the number of features is large compared to the number of observations in the datasets, this situation is commonly referred to as the "curse of dimensionality."). Thus, RF can handle larger datasets, both in dimension and attributes. The hybridized models outperformed their standalone counterparts. This enhanced performance occurred due to the hybridization which lead to a coupled model exhibiting higher flexibility, that is better trained and has a non-linear structure^70^. Given the non-linearity of the relationships between the variables and the weak connection between the individual variables and flow velocity, this flexibility and structure is particularly crucial for the forecasting of flow velocity.

Several factors explain why the hybrid M5P models outperformed all other hybridized models. First, M5P is a comparatively simple and interpretable algorithm, which makes the model's output simpler to comprehend and interpret. Second, M5P is capable of handling both continuous and categorical data, which is beneficial when working with datasets containing both categories of variables. Thirdly, M5P model contains two key components: growing stages and pruning stages. The growth stage involves splitting nodes based on the growth stage values of the characteristics, aiming to reduce the forecasting error for numerical responses at terminal nodes and increase the tree's depth. The pruning stage assesses the contribution of each attribute to a node's forecasting inaccuracy and subsequently prunes unnecessary branches. Fourthly, hybrid models that combine M5P with other algorithms can capitalize on the strengths of both models, resulting in a model that is more robust and accurate.

### Impact of input variables on the accuracy of model forecasting

The permutation of input variables significantly influenced the predictive capability of the model, underscoring that identifying the optimal combination is a crucial step in developing an accurate machine learning model. For instance, the input combination with variable "*h*_*v*_" removed exhibited over three times superior forecasting accuracy (in terms of NSE) compared to the least performing input combination. Consequently, a variety of input variable combinations must be explored during the optimization of machine learning models. We employed a manual approach in order to determine the optimal input combination. Methods like PCA and gamma test also provide for optimal input combination but they provide only one set of combination. Manually ascertaining the optimal combination can result in models with a superior forecasting performance because it is possible to determine the hyper-sensitivity parameters and comprehend the model's hyper-parameter reaction and trend by varying the input values.

The current paper showed that, in most of the cases, the optimal input combination corresponded to the inclusion of all the input parameters. Even parameters with low correlation with flow velocity, such as vegetation height and diameter, contributed to better forecasting power. This result further highlights the complex, nonlinear nature of the interaction of vegetation with flow mechanics, and the requirement for multiple input parameters to represent this interaction. Consequently, a variety of distinct input variable combinations must be taken into account during the optimization of machine learning models, even when channel, flow and vegetation parameters might a priori be considered ineffective.

### Capturing impact on flow velocity of vegetated alluvial channels through AI models

Vegetative elements significantly influence flow velocity in vegetated channels, and understanding these effects is critical for accurate forecasting and effective river management. Taller plants extend further into the flow, giving the water more surface area to push against, increasing drag force and decreasing flow velocity. Furthermore, dense canopies formed by taller vegetation greatly restrict water flow, increasing resistance and decreasing velocity, whereas shorter vegetation allows water to flow more freely, resulting in higher velocities. Taller vegetation contributes to more turbulence in the water column. The turbulence dissipates the water's kinetic energy, further reducing its velocity. In contrast, shorter plants have less surface area in contact with the water, resulting in less drag and faster water flow. In addition, shorter vegetation produces less turbulence, preserving the water's kinetic energy and maintaining a higher flow velocity.

These complex interactions between vegetation and flow mechanics demonstrate the nonlinearity of flow velocity in vegetated channels. Machine learning models, particularly hybrid models, have demonstrated great potential for capturing these complex nonlinear interactions. These models can learn the relationships between various vegetative parameters (such as height, density, and flexibility) and flow velocity by utilizing heterogeneous datasets. The superior performance of hybrid models demonstrates their ability to accurately forecast flow velocity in a variety of vegetative and channel conditions. The proposed AR-M5P model, for example, effectively integrates autoregressive components to account for temporal dependencies and combines them with tree-based algorithms to detect non-linear patterns in data.

This study compared twelve ML models, including hybrids like AR-M5P, to traditional empirical equations. The results showed that hybrid ML models outperformed empirical equations for predicting flow velocity in vegetated channels. These models excelled at accounting for the diverse and complex effects of vegetative elements on flow velocity. However, more research is needed to investigate how these models perform across a wider range of vegetation types and channel morphologies. Vegetation flexibility, spacing, and seasonal variations in vegetation characteristics all have an impact on model accuracy and should be taken into account in future studies.

### Applying machine learning methods to forecast flow velocity in vegetated channels

The results indicate that hybrid M5P models, particularly M5P models trained with an Additive Regression algorithm, have the potential to generate accurate forecasting of flow velocity in vegetated river channels. Such methods can be easily employed in regions/countries where understanding of the flow-vegetation processes in river systems is limited. The ML models developed in this paper offer primary advantages in terms of simplicity, ease of construction, and low operational costs. This stands in contrast to theoretical and numerical models, which frequently demand substantial prior knowledge and resources for their development. The main disadvantages are two-fold. In line with other statistical approaches, the models formulated in this research are tailored to the specific rivers under examination and employing them in different river settings might not produce comparable forecasting accuracy. The input parameter range will be wider than examined in this paper, despite using datasets composed from a variety of sources from both lab and field investigations. Thus, future studies should develop and apply ML models to rivers with differing channel and plant morphologies to test their wider applicability. Second, as a result of their 'black box' structure, these models have limited explanation regarding their results and are unable to provide insight into the physical factors that determine flow velocity.

The current study has considered seven controlling parameters, revealing that flow depth, channel slope, non-dimensional drag coefficient, height and the diameter of vegetation, and ratio of cylinders to vegetation per unit area must all be accounted for in ML models of flow velocity. Future studies should take into account how other characteristics, like vegetation flexibility and spacing, affect the effectiveness of these models where data is available. (e.g. Haslam^71^; Sand-Jensen^72^), assisting in identifying the key parameters influencing flow velocity and elucidating the reasons behind their variations among rivers characterized by distinct vegetation and channel properties.

### Applying hybrid ML models for forecasting natural issues

The AR-M5P algorithm, a hybrid approach combining autoregressive (AR) models with the M5P model tree, has demonstrated superior performance in our prediction tasks. This model’s effectiveness can be leveraged in several critical natural and environmental domains. AR-M5P can be used to model and predict climate variables such as temperature, precipitation, and sea-level rise. Its ability to handle both linear and non-linear relationships makes it particularly suitable for capturing the complex interactions inherent in climate systems. For instance, it can predict temperature anomalies or precipitation patterns, which are crucial for understanding and mitigating the impacts of climate change.

In order to estimate pan evaporation rates using meteorological data from three Iraqi stations, Elbeltagi et al.^73^ investigated the coupling of the additive regression model (AR) with four machine learning models including M5P. The AR-M5P model, which used wind speed, relative humidity, and minimum and mean temperatures, showed that hybrid methods can accurately predict complex hydrological relationships.

Elbeltagi et al.^74^ used five intelligent and hybrid metaheuristic machine learning algorithms (AR, AR-Bagging, AR-RandomSubspace, AR-M5P, and AR-REPTree) to predict monthly mean daily reference evapotranspiration using climatic data from two semi-arid regions in Pakistan (1987–2016). The results revealed that all models predicted monthly mean daily reference evapotranspiration with high precision, with the AR-M5P model achieving the highest accuracy.

The increasing need for agricultural production and frequent droughts require accurate estimation of actual evapotranspiration for effective irrigation management. Granata^75^ compared three machine learning models along with AR-M5P with different input variables to predict evapotranspiration using data from Central Florida. Vishwakarma et al.^76^ used the M5P model to assess dams' impact on river hydrology and daily water temperature in the Yangtze River at Cuntan, emphasizing the importance of accurate water temperature prediction for ecological and operational planning. These models offer dependable and cost-effective tools for forecasting water temperature, which helps with reservoir planning and environmental management.

In summary, the AR-M5P algorithm’s robustness and flexibility makes it a valuable tool for addressing a wide range of natural and environmental issues. Its ability to integrate and analyze multifaceted datasets allows for more accurate predictions and informed decision-making, ultimately contributing to the sustainability and resilience of natural systems. We trust that this enhanced discussion addresses your concern and illustrates the broader applicability of the AR-M5P model in tackling natural issues.

### Explainability of machine learning approaches used

#### Explainability of machine learning approaches in the context of AR-M5P

Explainability in ML refers to the ability to describe the inner workings and decision-making processes of models in a way that is understandable to humans. This is crucial for validating model predictions, ensuring user trust, and facilitating regulatory compliance. In the context of our study, the explainability of the AR-M5P algorithm can be discussed as follows:

#### Model structure and decision rules

The AR-M5P algorithm combines autoregressive models with M5P model trees, which are inherently more interpretable than many black-box models. The M5P model tree generates decision rules in the form of linear regression functions at its leaves. These rules can be easily inspected and interpreted to understand how the model makes predictions based on input features. For example, the decision paths in the tree can be traced to see how specific variables contribute to the final prediction.

#### Feature importance

The AR-M5P model provides insights into feature importance by indicating which variables are used in the decision nodes of the tree. By analyzing the frequency and impact of features at different nodes, we can identify the most influential variables driving the predictions. This helps in understanding the relative importance of each feature in the context of the model. Further, we have done input combinations in this study thereby incorporating the relative importance of features with respect to others. Also, we determine the sensitivity analysis to find the most influential parameter in this study.

#### Model simplification

While AR-M5P is more interpretable than many complex models, further simplification techniques, such as pruning the decision tree, can enhance interpretability without significantly compromising accuracy. Simplified models are easier to interpret and explain, making them more accessible to non-technical stakeholders.

In summary, the AR-M5P algorithm offers several avenues for explainability, from its inherently interpretable model structure. By leveraging these methods, we can enhance the transparency and interpretability of our ML predictions, thereby fostering greater trust and understanding among users and stakeholders.

### Limitations of the study

Predicting flow velocity in a vegetative alluvial channel can be quite challenging due to the numerous variables that require consideration. This study utilized a range of datasets from the literature, including the number of cylinders per unit vegetated area (N_v_), flow depth (D_f_), channel slope (α), vegetation height (h_v_), cylindrical vegetation diameter (d_v_), and non-dimensional drag coefficient (β_d_). However, various factors, such as the shape of the channel bed, the Froude number, the amount of water flowing through the channel, and more, can all impact the prediction of flow velocity. Our dataset was missing these factors, so our proposed methods did not take their influence into consideration. In addition, the range of variables plays a crucial role in the training of the ML method. Although our dataset includes data from various field and laboratory studies, there are instances where the input variables exceed the values considered by the authors. In these two instances, the proposed method may not perform as well as it currently does. These concerns are common in most ML-based methods, as training heavily depends on the dataset and its characteristics.

Gaussian noise is a key concept in signal processing and machine learning. It refers to a type of random variation that follows a Gaussian distribution. By injecting Gaussian noise into the data, we impose a level of unpredictability specified by this particular distribution. This has the potential to significantly alter the performance and analysis of the ML approach. In our method, we apply 10%, 20%, and 30% Gaussian noise to each column sequentially. This methodology introduces a specified level of disruption into the data, which might be useful for assessing the resilience and ability of ML approaches to apply to fresh data sets. An investigation of the influence of Gaussian noise on ML method performance frequently includes analyzing the ML method's ability to handle noisy inputs and determining whether it can still create correct predictions despite the increased variability (Table 6).

18 $$IA = 1 - \frac{{\sum\limits_{i = 1}^{N} {\left( {V_{i} - \widehat{V}_{i} } \right)^{2} } }}{{\sum\limits_{i = 1}^{N} {\left( {\left| {V_{i} - \overline{V} } \right| + \left| {\widehat{V}_{i} - \overline{V} } \right|} \right)^{2} } }},\quad 0 \le IA \le 1$$

**Table 6:** Gaussian noise added in input parameters by 10%, 20%, and 30% then performance analysis with best method (AR-M5P).

% Added Gaussian noise	Index of agreement (IA)
0	0.988
10	0.872
20	0.831
30	0.820

## Conclusion and future work

The precise forecasting of flow velocity in vegetated channels is important for estimating flooding and sediment transport. As a result of the non-linear interactions between vegetation and flow mechanics, machine learning methods have great potential for forecasting flow velocity with high accuracy. Using flow velocity measurements in natural and laboratory flume experiments, this research evaluated the performance of twelve ML models (Kstar, AR-Kstar, BA-Kstar, M5P, AR-M5P, BA-M5P, REPT, AR-REPT, BA-REPT, RF, BA-RF, AR-RF) for forecasting of flow velocity in an alluvial channel with submerged vegetation. Their performance was compared against those of four empirical equations, using a large number of datasets available in the literature. The main findings were as follows:

1. Results from a sensitivity analysis indicated that the most influential factor on flow velocity was vegetation height, followed by flow depth, the ratio of cylinders to vegetation per unit area, channel slope, non-dimensional drag coefficient, and vegetation diameter.
2. The AR-M5P model had the greatest predictive ability. According to Nash–Sutcliffe Efficiency values, all machine learning models displayed ‘very good’ performance and outperformed empirical models which had ‘unsatisfactory’ performance. All models, except two empirical equations, underestimated flow velocity.
3. Compared to standalone machine learning and empirical models, hybrid models have a superior forecasting power because they are more flexible in their internal structure and had capabilities of reproducing nonlinear interactions between vegetation, channel, and flow characteristics more effectively.
4. Nearly all ML methods performed accurately when all input parameters were utilized in model construction. Input variables exhibiting low correlation coefficients with flow velocity were found to enhance the accuracy of forecasting. As a result, the optimization of machine learning models necessitates the consideration of a diverse array of input variable combinations.

These results of this study shows that hybrid ML models possess tremendous potential in forecasting flow velocity and examining non-linear flow-vegetation interactions, particularly in situations where the physical processes under consideration are not fully understood. Consequently, understanding this potential over a wider range of vegetation and channel morphologies, and considering how other factors affect the performance of these models, such as vegetation flexibility and spacing, is a crucial research avenue for river scientists.

Future work on flow velocity prediction in vegetated channels could explore and improve in a number of directions. Firstly, the ML method could enhance its ability to capture non-trivial data by incorporating cutting-edge deep learning architectures such as convolutional neural networks (CNNs) or recurrent neural networks (RNNs). Domain-specific properties related to fluid dynamics and hydrodynamics may enhance the prediction capacity of the ML approach. As a result, predictions for different flow patterns, vegetation types, and scenarios may be more accurate and reliable. Also, studying hybrid machine learning methods that combine data-driven machine learning methods with physics-based ML techniques could combine the benefits of both approaches, making predictions more accurate without sacrificing the ability to understand how physical things work. Furthermore, the acquisition of a larger and more diverse dataset encompassing a wide range of flow conditions, geometries, and sizes facilitates the training of machine learning algorithms that can handle real-world scenarios with greater precision and reliability. Furthermore, the development of easy-to-use software tools or platforms for predicting flow velocity in vegetated channels using web-based techniques or Android apps can improve their acceptability. This way, we can practically implement our work on river flow management and environmental protection.

## Data Availability

Data will be made available on request from the corresponding author Vishal Deshpande at  deshpande@iitp.ac.in .

## List of symbols

*V*: Flow velocity
*N*_*v*_: Number of cylinders per unit vegetated area
*D*_*f*_: Flow depth
*α*: Channel slope
*h*_*v*_: Height of the vegetation
*d*_*v*_: Diameter of cylindrical vegetation
*β*_*d*_: Non-dimensional drag coefficient
*g*: Gravitation acceleration
